# Case Report: Distinctive EEG Patterns in SCARB-2 Related Progressive Myoclonus Epilepsy

**DOI:** 10.3389/fgene.2020.581253

**Published:** 2020-12-03

**Authors:** Mostafa Hotait, Maya Dirani, Tarek El Halabi, Ahmad Beydoun

**Affiliations:** Department of Neurology, American University of Beirut Medical Center, Beirut, Lebanon

**Keywords:** SCARB2, progressive myoclonus epilepsy, action myoclonus with renal failure (AMRF), fixation-off phenomenon, EEG

## Abstract

Action myoclonus-renal failure syndrome (AMRF) is a rare, recessively inherited form of progressive myoclonus epilepsy (PME) caused by mutations in the SCARB2 gene and associated with end-stage renal failure. In addition to severe progressive myoclonus, the neurological manifestations of this syndrome are characterized by progressive ataxia and dysarthria with preserved intellectual capacity. Since its original description, an increasing number of “AMRF-like” cases without renal failure have been reported. We describe the case of a 29-year-old woman with progressive disabling myoclonus associated with dysarthria and ataxia who was found to have a novel homozygous frameshift mutation in the SCARB2 gene. In addition, this report emphasizes the presence of two EEG patterns, fixation-off phenomenon, and bursts of parasagittal spikes exclusively seen during REM sleep that appear to be characteristic of this condition.

## Introduction

The progressive myoclonus epilepsies (PME) consist of a group of genetic neurodegenerative disorders that accounts for <1% of all epilepsies (Bhat and Ganesh, 2018). They are characterized by a distinctive triad of progressive myoclonus, epileptic seizures, and neurologic deterioration. Most PME are inherited in an autosomal recessive manner and can be distinguished according to age at symptom onset and the predominant neurological abnormalities, particularly dementia, dysarthria, or ataxia (Scala et al., 2020).

Action myoclonus-renal failure syndrome (AMRF, OMIM 254900) is a rare, autosomal recessive form of PME associated with end-stage renal failure requiring dialysis or transplantation. This condition, first described by Andermann et al. in four French-Canadian patients (Andermann et al., 1986) was found in 2008 to be caused by mutations in the SCARB2 gene (Berkovic et al., 2008). SCARB2 encodes for a lysosomal membrane protein essential for maintaining endosomal transport and lysosomal biogenesis (Eskelinen et al., 2003). This syndrome is characterized by a progressive disabling myoclonus frequently associated with ataxia and dysarthria and preserved intellectual capacity (Hopfner et al., 2011). Its clinical features overlap with those of Unverricht-Lundborg disease (ULD), another PME resulting from mutations in the gene encoding cystatin B (CSTB). In fact, in a genetic study of 41 patients with clinical features suggestive of ULD but with no mutations in the CSTB gene, five patients were found to have a SCARB2 mutation (Dibbens et al., 2009).

We report a novel SCARB2 frameshift mutation in a patient with progressive disabling action myoclonus without renal failure and suggest that this condition is associated with characteristic EEG patterns.

## Case

A 29-year-old, left-handed Iraqi woman with normal neurodevelopment, born to first-degree cousins and a member of a dizygotic twin pregnancy, presented for refractory epilepsy. At the age of 19 years, she started experiencing spontaneous and action-induced myoclonus, which were often precipitated by external stimuli, predominantly light sources. One year later, she experienced a nocturnal bilateral tonic-clonic (BTC) seizure for which she was started on levetiracetam. Over the next 2 years, the BTC seizures abated along with improvement in the frequency and severity of the myoclonic jerks. Subsequently, the BTC seizures recurred and were refractory to different combinations of levetiracetam, valproate, and clonazepam. Her myoclonic jerks progressively worsened and were exacerbated by light sources, which resulted in her spending most of her time in darkened rooms. At the age of 27, the patient developed progressively worsening dysarthria and disabling ataxia.

Her neurologic examination was relevant for severe dysarthria with barely comprehensible speech, a prominent tremor and frequent spontaneous and action myoclonus. In addition, she was severely ataxic and could not ambulate without assistance. Her cognitive function, motor strength, and muscle tone were otherwise normal. An epilepsy protocol brain MRI revealed a mild generalized cerebral atrophy with no abnormalities detected on a post-acquisition voxel-based morphometric analysis.

**Figure 1:**
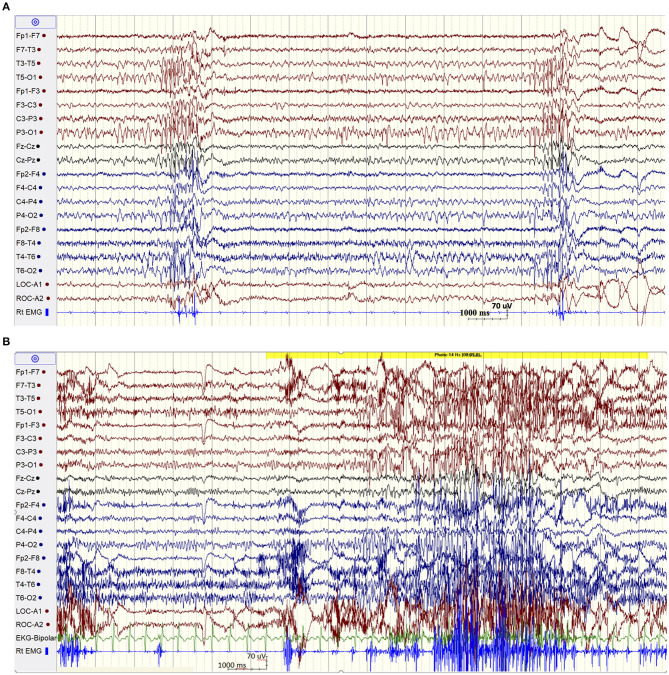
**(A)** Spontaneous bursts of repetitive spikes with an occipital predominance associated with myoclonic jerks. **(B)** EEG showing normal posterior background and a non-sustained photoparoxysmal response associated with multifocal myoclonic jerks following stimulation at 14 Hz.

**Figure 2:**
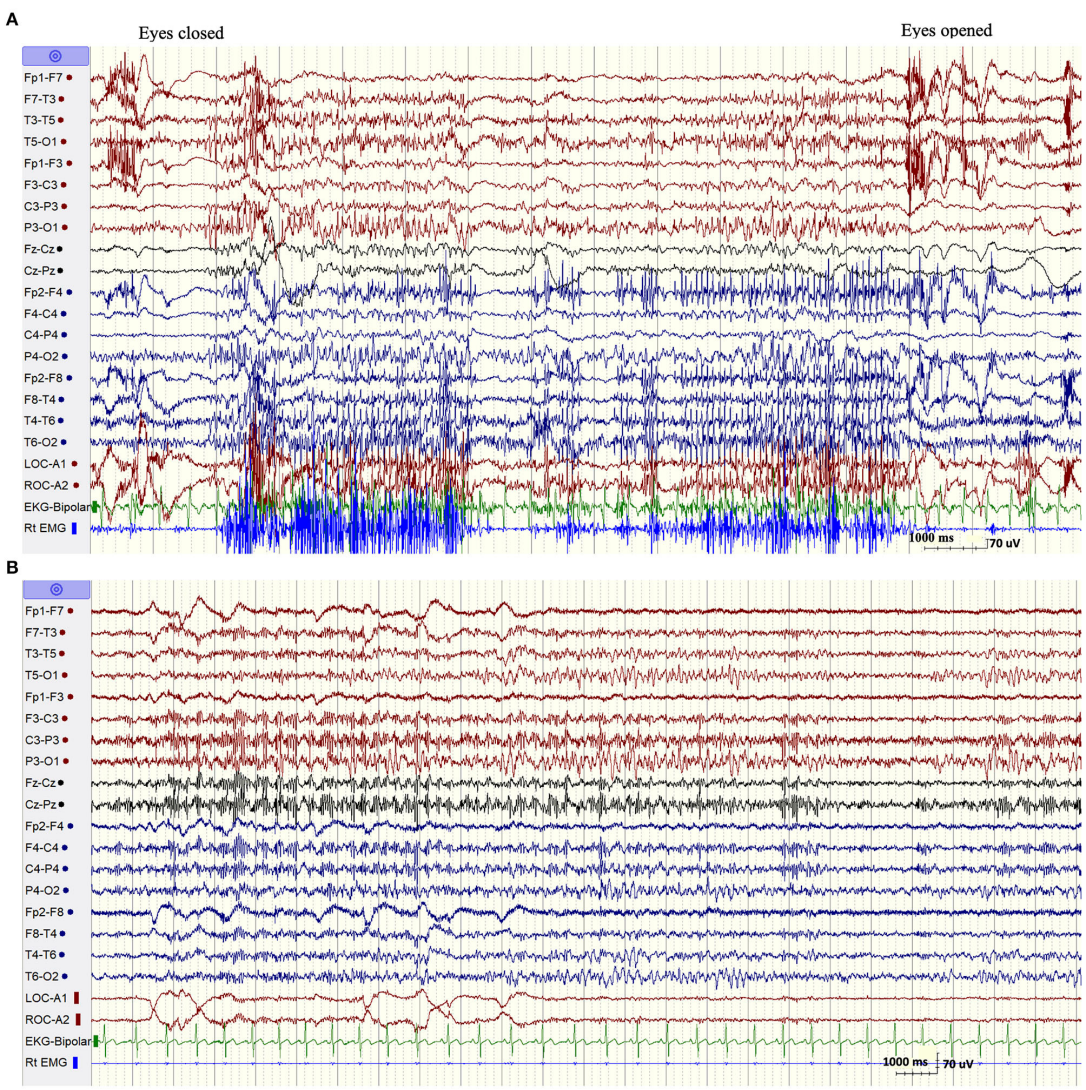
**(A)** Fixation off sensitivity. **(B)** REM sleep. A peculiar pattern consisting of repetitive spikes over the parasagittal derivations associated with fragmentary minimyoclonus of the right hand.

The patient was admitted for long-term video/EEG monitoring. Her EEG revealed a normal posterior background as well as frequent bursts and fragments of generalized spike and polyspike and wave discharges (GPSWD) with an occipital amplitude predominance, commonly associated with myoclonic jerks (Figure 1A). Intermittent photic stimulation at a frequency of 14 Hz triggered bursts of posteriorly predominant repetitive spikes that were associated with multifocal myoclonus, following which the photic stimulation was aborted (Figure 1B). Although the patient felt scared and uncomfortable following stimulation at flash frequencies of 2, 4, 6, 8, 10, and 12 Hz, those were not associated with a photoparoxysmal response or with worsening of her myoclonic jerks. In addition, a fixation-off phenomenon was consistently noted following eye closure and was characterized by bilateral posteriorly predominant repetitive spikes associated with violent multifocal myoclonus. The epileptiform discharges and associated myoclonic jerks persisted as long as her eyes remained closed and were immediately aborted upon eye opening (Figure 2A). In addition, the patient experienced frequent prolonged myoclonic seizures as well as a myoclonic-tonic-clonic seizure characterized by crescendo multifocal myoclonus that evolved to a BTC seizure. During REM sleep, a peculiar pattern consisting of bursts of repetitive parasagittal spikes associated with fragmentary minimyoclonus of the hands was seen (Figure 2B).

On whole exome sequencing (WES), a novel mutation of the SCARB2 gene was detected, c.40dup p. (Leu14Profs^*^35), creating a shift in the reading frame starting at codon 14 and ending in a stop codon 34 positions downstream. The WES and Sanger sequencing were validated by Centogene. Biochemical investigations showed a normal serum creatinine, and a 24-h urine collection revealed a normal creatinine clearance with elevated proteinuria at 51.1 mg/dL (normal range: 0–20 mg/dL). Perampanel was added to her treatment regimen and was titrated to 4 mg daily without any significant improvement in the frequency of her myoclonic jerks. Because this drug was not available in Iraq, the dose was not increased, and the medication was stopped due to lack of efficacy.

## Discussion

In this report, we present a patient diagnosed with AMRF based on a homozygous frameshift mutation in the SCARB2 gene resulting in SCARB2 deficiency. This variant is novel and was not identified in the NHLBI Exome Sequencing Project (ESP) Exome Variant server (http://evs.gs.washington.edu/EVS).

Since its original description in 1986 (Andermann et al., 1986), a total of 27 cases of AMRF from 20 families have been reported (Tian et al., 2018). Although its name implies the development of renal failure, typically within 0–5.5 years following symptom onset, 13 of the 27 reported cases did not have evidence of renal dysfunction despite being followed for up to 15 years until death (Dibbens et al., 2009; Tian et al., 2018). Our patient did not have evidence of renal insufficiency 10 years into her illness although we did document the presence of proteinuria on a 24-h urine collection following the results of the genetic testing. In light of the increased number of “ULD-like” cases without renal involvement, it is becoming clear that renal failure is not an invariable accompaniment of PME due to SCARB2 mutations. Because the phenotypes of the classical AMRF and those without renal failure are otherwise indistinguishable (Tian et al., 2018), it is best to refer to this condition as a SCARB2-related PME.

The EEG findings in SCARB2-related PME have been overall poorly documented with the exception of one previous study (Rubboli et al., 2011). The abnormalities included a progressively slowed background with irregular theta and delta activity intermixed with alpha activity and associated with intermittent bursts of GSWD (Tian et al., 2018). However, we noticed two distinctive EEG patterns that appear to be characteristic of this condition and which were not previously emphasized (Rubboli et al., 2011; Tian et al., 2018). The first pattern consisted of eyes-closed/fixation-off sensitivity (FOS), a phenomenon induced by elimination of central vision and characterized by the appearance of focal occipital or generalized epileptiform discharges following eye closure and persisting as long as the eyes are closed (Koutroumanidis et al., 2009; Brigo et al., 2013). Our patient consistently experienced intense multifocal myoclonic jerks with her eyes closed. These were associated with biposterior bursts of repetitive spikes that immediately aborted following eye opening. FOS is a rare phenomenon, most commonly encountered in children with the occipital epilepsy of Gastaut and the Panayiotopoulos syndrome, and anecdotally described in other focal or generalized epilepsy (Koutroumanidis et al., 2009; Brigo et al., 2013). It was, however, previously reported in two patients with SCARB2-related PME (Rubboli et al., 2011), but not in other types of PME with the exception of a single case of myoclonic epilepsy with red ragged fibers (Garcia Silva et al., 1987). More importantly, it has never been described in ULD, the condition resembling SCARB2-related PME at onset. In one patient with Lafora disease, the intractable myoclonic seizures were actually suppressed by fixation-off. In fact, neurophysiologic studies documented that fixation was the most important enhancer of myoclonus in this condition (Kumada et al., 2006). This is the opposite of what occurs in fixation-off sensitive epilepsies, a phenomenon described as inverted fixation-off sensitivity (Kumada et al., 2006).

The second EEG finding consisted of the exclusive appearance during REM sleep of bursts of bilateral repetitive polyspikes over the parasagittal derivations associated with subtle fragmentary myoclonus of the extremities. This pattern, initially described by Tassinari in 10 of 14 patients with ULD (Tassinari et al., 1974), was confirmed by other investigators and was believed to be one of the diagnostic clues of this disease (Ferlazzo et al., 2007; Avanzini et al., 2016). Our finding, which was also previously recorded on two patients with SCARB2-related PME (Rubboli et al., 2011), indicates that it is not specific for ULD but can also be seen in SCARB-2-related PME. Although those two EEG patterns are quite rare, their specificities in allowing distinguishing between the various forms of PMEs are yet undetermined. However, in the correct clinical setting, those EEG findings, especially the substantial worsening of myoclonus with eyes closed, should raise the suspicion of a SCARB-2-related PME.

We elected to add perampanel to the treatment regimen of our patient based on a previous positive experience with this drug in the treatment of Lafora disease and ULD (Dirani et al., 2014; Goldsmith and Minassian, 2016; Crespel et al., 2017). Although our patient did not titrate the perampanel dose beyond 4 mg daily, she did not notice any improvement while on this drug. The efficacy of perampanel needs to be tested in a cohort of patients before any conclusive determination of its value in SCARB-2-related PME.

We describe the third case of a patient of Arab descent to be diagnosed with a novel homozygous frameshift mutation of the SCARB-2 gene. This report emphasizes the presence of two EEG patterns, FOS and bursts of parasagittal spikes, exclusively seen during REM sleep that appear to be characteristic of this condition. Further studies are needed to confirm those findings.

## Data Availability Statement

The raw data supporting the conclusions of this article will be made available by the authors, without undue reservation.

## Ethics Statement

Ethical approval was not provided for this study on human participants because this is a case report that does not require an ethical approval at our institution. The patients/participants provided their written informed consent to participate in this study. Written informed consent was obtained from the individual(s) for the publication of any potentially identifiable images or data included in this article.

## Author Contributions

MH and MD followed the patient clinically and were responsible for initial manuscript writing. TE reviewed the literature. AB was responsible for the final version of manuscript. All authors contributed to the article and approved the submitted version.

## Conflict of Interest

The authors declare that the research was conducted in the absence of any commercial or financial relationships that could be construed as a potential conflict of interest.
